# Parental Revenge Bedtime Procrastination and Parent–Child Feeding Interactions in Early Childhood: A Theory-Driven Perspective on an Unstudied Pathway

**DOI:** 10.3390/clockssleep8040063

**Published:** 2026-09-30

**Authors:** Silvia Cimino, Luca Cerniglia

**Affiliations:** 1 Department of Dynamic, Clinical, and Health Psychology, Sapienza University of Rome, 00136 Rome, Italy; 2 Faculty of Psychology, International Telematic University Uninettuno, 00186 Rome, Italy; luca.cerniglia@uninettunouniversity.net

**Keywords:** bedtime revenge procrastination, parent–child interactions, feeding, emotional dysregulation

## Abstract

Bedtime procrastination is defined as delaying sleep despite having no external obstacles. It is a well-established marker of impaired self-regulation and contributes to sleep loss, daytime fatigue, and emotional dysregulation. A parental variant of this behavior is termed parental revenge bedtime procrastination (RBP). This behavior is conceptualized as an autonomy-motivated subtype in which caregivers reclaim personal time at night to compensate for daytime caregiving demands. However, parental RBP has received almost no direct empirical attention, particularly regarding its impact on parent–child interaction quality. Concurrently, distinct literatures on parental technoference, sensitivity, and responsive feeding converge on a shared theme. A sleep-deprived, depleted, and phone-absorbed parent may struggle to read and respond to a young child’s hunger and satiety cues during meals. Grounding these relational dynamics in classical infant mental health frameworks highlights the developmental stakes of impaired mealtime reciprocity. Key theoretical foundations include Daniel Stern’s theory of affective attunement, Infant Research paradigms by Beebe and Tronick, contemporary neurocognitive models, and Irene Chatoor’s clinical classification of feeding disorders. To our knowledge, no study has tested this direct chain in children aged 0–3 years. This perspective article maps existing evidence onto a conceptual model linking parental RBP to feeding interaction quality. It identifies empirically supported segments versus inferential links, proposes a concrete research agenda, outlines potential clinical implications, and advocates for the systematic investigation of this pathway.

## 1. Introduction: The Gap

Two extensively studied literatures rarely intersect. The first concerns bedtime procrastination as a self-regulation failure, supported by robust evidence linking it to shorter and poorer-quality sleep, greater daytime fatigue, and lower self-control [1,2,3,4]. The second concerns parental responsiveness during feeding, where feeding style—largely a function of parental responsiveness and demandingness—shapes whether a young child develops adaptive eating self-regulation or comes to rely on external, non-hunger cues to eat [5,6]. What is missing is any empirical study connecting the two: an examination of whether parents who engage in revenge bedtime procrastination—using the night to reclaim autonomy after a depleting day of caregiving—subsequently show measurable differences in the quality of their feeding interactions with infants and toddlers.

To verify the existence of this gap, a targeted narrative search was conducted across PubMed, PsycInfo, Scopus, and Google Scholar using combinations of search terms for bedtime procrastination (“bedtime procrastination”, “sleep delay”, “revenge bedtime procrastination”) alongside parenting and feeding constructs (“parenting”, “parental sensitivity”, “technoference”, “infant feeding”, “responsive feeding”, “feeding interactions”). This targeted search suggested that while isolated links between sleep loss, parental stress, and general parenting quality exist, no empirical study to date has evaluated parental revenge bedtime procrastination as an upstream predictor of early feeding interaction quality.

This absence is notable because the mechanistic building blocks for such a pathway already exist in the literature, scattered across sleep, technoference, coparenting, and feeding research. Furthermore, early feeding is widely recognized by developmental psychologists as one of the primary arenas for parent–child relational regulation, mutual affect sharing, and autonomic alignment. Focusing specifically on the early developmental period of 0–3 years is critical, yet this age bracket encompasses marked developmental shifts. The pathway must be understood across exclusive milk feeding in early infancy (where interaction centers on physiological regulation and attachment security), complementary feeding (negotiating novel textures and sensory inputs), and toddler self-feeding (where mealtimes become a primary arena for autonomy negotiation and boundary setting). Parental fatigue and impaired responsiveness may manifest differently across each of these distinct developmental phases.

To understand why parental fatigue and bedtime delay might disrupt feeding interactions, one must integrate foundational developmental frameworks. Daniel Stern’s theory of Affective Attunement [7] focuses on the subjective matching of emotional states and temporal rhythms during micro-interactions, explaining why moment-to-moment parental attunement forms the foundation for infant internal working models. Beebe and Tronick’s Dyadic Regulation framework [8,9] provides micro-analytic evidence of split-second contingent responsiveness, gaze synchronization, and repair of relational mismatches, illustrating how parental distraction or fatigue physically interrupts dyadic co-regulation. Irene Chatoor’s Feeding Framework [10,11] translates developmental attunement into clinical feeding contexts, explaining where impaired parental responsiveness disrupts the child’s internal somatic awareness of hunger and satiety, heightening the risk of conflictual feeding routines. Finally, contemporary neurocognitive models [12,13] bridge these classical theories with modern psychophysiology, demonstrating how parent–child co-regulation relies on the alignment of attentional and autonomic systems.

This perspective assembles these building blocks—from classical infant mental health theory and contemporary dyadic developmental science to modern sleep and technoference research—into an explicit, testable model and evaluates, segment by segment, how much support currently exists for each proposed link.

## 2. Parental Revenge Bedtime Procrastination as a Self-Regulation Phenomenon

Bedtime procrastination is not primarily a problem of poor sleep hygiene knowledge; it is best characterized as a failure of self-regulation under conditions of depletion. Meta-analytic and cross-sectional evidence links it consistently to lower self-control and lower trait self-regulation [1,2,3], and it is associated with shorter sleep duration, poorer subjective sleep quality, and greater next-day fatigue [2,3]. Recent work extends this to psychological distress, describing bedtime delay as a stress-worsened self-regulation failure pursued for leisure despite its costs [4]. However, the vast majority of existing empirical studies have been conducted on university students, young adults, or general population samples; direct empirical data specifically targeting parents of infants and young children remain extremely sparse.

Furthermore, parental revenge bedtime procrastination (RBP) must currently be treated as a proposed autonomy-motivated subtype of bedtime procrastination that requires formal psychometric validation. The incremental scientific value of proposing parental RBP lies in testing whether the specific psychological drive to reclaim autonomy (“me-time”) after caregiving depletion predicts unique variance in daytime caregiving behavior beyond established constructs—such as general bedtime procrastination, total sleep loss, caregiving overload, general self-control failure, or problematic smartphone use. Until empirical studies demonstrate this incremental predictive validity, RBP should be conceptualized as a specialized subtype rather than an entirely independent construct.

To distinguish parental RBP from overlapping phenomena, it must be clearly delineated from general bedtime procrastination, primary insomnia, shift work, necessary nighttime labor, circadian chronotype preferences, and child-driven night awakenings. Operatively, parental RBP involves voluntary sleep delay occurring despite an available sleep window, driven by a subjective drive to reclaim personal autonomy following daytime caregiving exhaustion, and occurring in the absence of direct child-induced obstacles. Crucially, voluntary bedtime delay leads to actual sleep loss only under specific structural conditions—namely, when the parent cannot recover lost sleep by sleeping later in the morning or taking naps during the day.

It is critical to differentiate the observable behavior (delaying bedtime) from the underlying psychological motive (reclaiming autonomy). Standard bedtime procrastination measures assess late-night sleep delay but do not capture the specific compensatory drive characteristic of RBP. Moreover, while self-regulation depletion models suggest executive function is depleted by late evening, alternative theoretical mechanisms must also be recognized, including habit strength, circadian eveningness preferences, stress avoidance, delay discounting, and immediate reward seeking through digital media. Critically, late-night device use among parents is non-uniform; using a smartphone at night to seek parenting support, communicate with family, or manage household logistics represents functional recovery or utility rather than RBP. Finally, voluntary bedtime delay must be rigorously separated from externally imposed sleep loss. Parents of infants frequently experience severe sleep disruption caused directly by child night awakenings, illness, or feeding needs. While both forms yield daytime fatigue, voluntary RBP stems from autonomy depletion, whereas infant-driven sleep loss stems from direct caregiving execution, carrying distinct psychological antecedents and intervention targets.

## 3. From Delayed Sleep to Caregiving Quality: The Technoference Pathway

The most direct empirical anchor for the model’s transition from sleep loss to caregiving behavior comes from research on parental smartphone use. In parents of infants, heavier bedtime phone use clusters with poorer sleep and higher depressive symptoms [14], suggesting that device use implicated in nighttime leisure is embedded in broader sleep disruption. Critically, this device use does not stay confined to the night. Co-present parental smartphone use during daytime caregiving—termed technoference—is common and has been documented across routine parenting contexts, including mealtime [15].

However, the directional sequence between RBP, sleep loss, and daytime technoference remains a theoretical hypothesis rather than a proven causal path. Technoference should be conceptualized flexibly within the model: while late-night device use during RBP may directly reinforce daytime device habits as a downstream consequence, daytime technoference can also act as an antecedent habit or a parallel correlate driven by underlying self-regulation depletion, smartphone addiction, or psychological distress. Competing temporal sequences exist: pre-existing psychological distress, postpartum depression, or problematic smartphone habits could independently drive both late-night scrolling and daytime media absorption.

Furthermore, parental device use during caregiving varies significantly in duration, timing, purpose, and level of cognitive absorption. Briefly checking a feeding-tracking application or responding to an urgent message carries vastly different relational consequences than becoming deeply absorbed in social media feeds during a meal. High cognitive absorption poses the primary threat to parental sensitivity.

During infant feeding, deep parental device absorption is associated with reduced gaze toward the infant and altered parental physiological arousal [15], and scoping reviews confirm links between screen use and reduced parental responsiveness [15]. While this dynamic shares conceptual similarities with Tronick’s [9] Still-Face Paradigm, important limits to this analogy must be acknowledged: natural, intermittent smartphone glances during mealtimes do not equal the complete, experimentally forced freeze of facial affect in the Still-Face protocol, and claims of equivalent developmental harm should be avoided.

Finally, the empirical literature in this domain relies heavily on maternal samples. Studies documenting reduced gaze, facial immobility, and physiological arousal during feeding have predominantly evaluated mothers. Care must be taken not to generalize maternal findings to fathers or alternative caregivers without direct empirical verification.

## 4. From Caregiving Quality to Feeding Interaction Outcomes

The model’s transition from reduced parental responsiveness to feeding-specific outcomes is grounded in the feeding-styles literature. Feeding styles are explicitly defined along the dimensions of responsiveness and demandingness, so any reduction in parental responsiveness has direct relevance to the quality of feeding interactions [5]. It must be emphasized that existing feeding-style studies validate the relationship between parental responsiveness and child eating self-regulation, but they have not examined parental sleep behavior or RBP as the upstream predictor.

When mapping potential outcomes, it is essential to distinguish between immediate interactional outcomes (such as reduced mealtime gaze, missed hunger and satiety cues, and temporal misattunement), intermediate child behavioral responses (including mealtime distress, active food refusal, and heightened distraction), and long-term developmental trajectories (such as altered child interoceptive awareness, persistent pediatric feeding difficulties, or altered growth patterns).

Consistent with this, lower parental awareness of a child’s internal eating cues is associated with poorer eating self-regulation [6]. Building upon Chatoor’s clinical framework [10,11]—which operates alongside modern multi-factorial systems such as the Pediatric Feeding Disorder (PFD) framework [16]—we hypothesize that depleted, sleep-deprived parents may vacillate between two tentative interaction patterns: an inattentive/disengaged pattern, where cue-reading is absent due to cognitive exhaustion or device distraction, and an intrusive/coercive pattern, where fatigued parents force-feed or rush the meal to end it quickly.

However, severe clinical outcomes—such as chronic food refusal, growth faltering, or pediatric feeding disorders—are highly complex and multi-determined. They involve biological, oral-motor, sensory, medical, and environmental factors. Parental fatigue and altered responsiveness should be framed as potential relational contributors within a broader multi-factorial matrix, rather than primary isolated causes.

## 5. The Missing Direct Link

The weakest segment of the model is also its most novel contribution: the path from parental sleep-related self-regulation failure to specific child feeding outcomes. A recent systematic review reports that evidence remains mixed or insufficient when the predictor is early sleep or emotion regulation rather than feeding behavior itself [17,18,19]. Calling parental RBP an “originating cause” of child feeding difficulties is unwarranted; it is strictly a hypothesized upstream contributor.

To evaluate whether this direct link truly exists, future empirical studies must implement rigorous statistical controls for key confounding variables, including parental mental health (depressive and anxiety symptoms), pre-existing problematic device use, child temperament (negative affectivity, sensory sensitivities), child medical/feeding conditions, pediatric sleep disruption, socioeconomic stress, and household routines.

Furthermore, researchers must test explicit, falsifiable hypotheses, for example: does parental RBP predict observed mealtime responsiveness and dyadic synchrony after controlling for total sleep duration, parental depression, and child night-waking frequency? If RBP fails to account for unique variance over and above total sleep loss and depressive symptoms, the specific utility of the construct in this pathway would be falsified. Finally, a complete theoretical model must incorporate bidirectional, child-to-parent feedback loops. Infant characteristics—such as difficult temperament, sensory food aversion, chronic night waking, or medical vulnerabilities—exert immense caregiving strain on parents. A dysregulated or food-refusing infant heightens daily parental exhaustion and reduces perceived daytime autonomy, which in turn may directly escalate the parent’s nighttime motivation to engage in compensatory RBP.

## 6. Moderators of the Proposed Pathway

The proposed relationship will not operate identically across all families. A range of family, structural, and individual factors may buffer or intensify the hypothesized pathway. Rather than treating these variables as established statistical moderators, they should be understood as proposed contextual and protective factors whose specific location and direction of effect within the cascade require empirical testing.

At the individual level, higher parental general self-regulation capacity helps caregivers maintain consistent feeding routines despite fatigue [20,21], buffering the link between sleep loss and reduced responsiveness. At the interpersonal level, high coparenting quality—defined as executive coordination and solidarity between partners—mitigates autonomy strain and reduces the motivation for late-night RBP [22,23]. Similarly, active direct caregiving involvement by fathers or non-carrying partners provides structural relief during high-demand parenting periods [24,25,26], directly reducing primary caregiver depletion.

Crucially, macro-structural and socioeconomic factors must be integrated into the model. Structural stressors—including low income, single parenthood, shift work, housing instability, lack of affordable childcare, and the absence of extended family support—severely deplete parental bandwidth independent of individual self-control capacities. These structural pressures amplify autonomy strain and cumulative daily fatigue. Finally, the establishment of explicit digital boundaries—defined as device-free mealtime rules, nocturnal disconnection limits, and the parental capacity for intentional disengagement [15]—is hypothesized to interrupt the transition from fatigue to caregiving technoference.

## 7. The Proposed Conceptual Model

The model explicitly separates the primary core sequence from secondary extensions, contextual buffers, and child-driven feedback loops.

The model posits a specific temporal ordering of parallel mediators: late-night RBP (the defining behavior) leads to acute sleep loss (provided sleep cannot be recovered by waking later or napping), generating morning cognitive depletion and fatigue, which impairs emotion regulation, thereby heightening susceptibility to daytime device absorption (technoference) and lowering contingent responsiveness during mealtimes. In addition, a bidirectional feedback loop operates continuously, as stressful, conflictual child feeding interactions increase daily parental emotional exhaustion, reinforce the perceived loss of daytime autonomy, and escalate the late-night drive for compensatory RBP.

To provide complete conceptual precision, Table 1 delineates each proposed relationship within the conceptual model, specifying the proposed direction, current level of evidence, primary confounding variables to control, and alternative competing explanations.

## 8. Evaluation of Evidence Strength

To ensure scientific clarity, the proposed model’s strength is evaluated against explicit criteria: Strong (multiple consistent empirical studies in parent populations with direct measures), Moderate (consistent evidence in general/adult populations or indirect parenting measures), and Weak/Hypothetical (theoretical extrapolation without direct empirical testing).

Rather than evaluating the overall model as a single monolith, each proposed segment must be evaluated independently:

- RBP leading to curtailed sleep (Link 1): Moderate evidence. Supported by general adult, student, and population studies [1,2,3,4], but direct empirical validation in parents of infants remains sparse. Crucially, this link holds only when structural constraints prevent morning sleep recovery.
- Curtailed sleep leading to next-day cognitive or emotional difficulties (Link 2a): Strong evidence. Supported by the extensive neurocognitive and psychophysiological literature on executive function depletion following sleep restriction.
- Cognitive/emotional difficulties affecting parental responsiveness (Link 2b): Moderate evidence. Supported by studies linking parental burnout, exhaustion, and executive dysfunction to reduced parental warmth and sensitivity.
- Nighttime device use predicting daytime technoference (Link 2b alternative/correlate): Moderate evidence. Supported by observational studies on device habits [14,27], though directionality remains unproven (technoference can be an antecedent or parallel habit rather than a pure downstream outcome).
- Technoference affecting feeding-specific responsiveness (Link 3): Moderate-to-Strong evidence. Supported by direct observational studies demonstrating that device-absorbed parents miss infant cues and show reduced gaze/affect matching during mealtimes [15].
- Parental responsiveness affecting child feeding outcomes (Link 4): Strong evidence. Validated extensively within the responsive feeding and pediatric feeding styles literature [5,6,16].
- The overall sequential chain from parental RBP to child feeding outcomes (Link 5): Weak and Hypothetical. No empirical study has tested this complete pathway longitudinally or cross-sectionally. Demonstrating individual components does not prove they operate in a sequential causal chain. Classical frameworks (Stern, Beebe, Chatoor) provide theoretical plausibility, not empirical proof.

## 9. Potential Clinical Research & Prevention Frameworks

Because the link between parental RBP and child feeding outcomes remains an untested hypothesis, all clinical implications must be understood as theoretical concepts for future research rather than current practice guidelines.

At the population level, public health messaging could explore non-judgmental, structural approaches that normalize autonomy needs. Late-night leisure seeking might be conceptualized as an understandable response to caregiving depletion rather than an individual moral or self-control failure. Furthermore, public health initiatives could investigate whether systemic work–life balance supports—such as affordable childcare, paid parental leave, and flexible work hours—preserve daytime personal time for parents and subsequently impact caregiving quality.

In clinical research settings evaluating pediatric feeding difficulties, multidisciplinary protocols may explore screening for parental rest without jumping to causal conclusions. Before examining relational dynamics, clinicians and researchers must ensure that child food refusal is thoroughly evaluated for medical, oral-motor, sensory, nutritional, and organic causes. In addition, exploratory screening tools could be tested to differentiate primary insomnia, infant-driven sleep loss, and voluntary RBP.

Video-feedback coaching (derived from Beebe [8]) represents a candidate intervention target to be empirically evaluated specifically for sleep-deprived caregivers struggling with mealtime attunement to test whether improving cue-reading buffers against fatigue-related detachment. Crucially, clinical research frameworks must be inclusive of diverse family structures. Recommendations must extend beyond traditional coparenting models; for single parents, shift workers, or families experiencing financial strain, future research should investigate the protective role of community support networks, extended family assistance, and flexible daily pacing rather than assuming a co-resident partner is available to absorb evening duties [28,29,30,31,32,33,34,35,36,37,38].

## 10. Future Directions: A Staged Empirical Research Roadmap

To rigorously evaluate the proposed model, future research should follow a sequential, five-stage empirical roadmap:

- Stage 1 (Qualitative & Construct Development): Conduct qualitative interviews with diverse parents to explore how nighttime autonomy seeking is experienced, establishing initial construct boundaries.
- Stage 2 (Psychometric Validation): Develop and validate a Parental Revenge Bedtime Procrastination Scale (P-RBPS), demonstrating factor structure, internal consistency, and incremental validity over standard bedtime procrastination, general sleep loss, and problematic smartphone use scales.
- Stage 3 (Daily Process & EMA Studies): Employ 14-to-21-day Ecological Momentary Assessment (EMA) combined with wrist actigraphy and passive smartphone logging to capture real-time within-parent fluctuations among daily caregiving stress, nocturnal RBP, sleep loss, and next-day fatigue.
- Stage 4 (Observational Mealtime Micro-Analysis): Video-record parent–child feeding interactions across milk, complementary, and toddler self-feeding stages, micro-coding gaze, affect matching, temporal lagging, and technoference interruptions.
- Stage 5 (Longitudinal & Multilevel Modeling): Execute multi-wave longitudinal designs tracking parent–child dyads from infancy to age 3, testing whether within-parent variations in RBP predict longitudinal trajectories of child eating self-regulation, while controlling for parental mental health, child temperament, and structural stress.

Future empirical studies must prioritize diverse samples across socioeconomic statuses, single- and dual-parent households, varied caregiver genders (actively recruiting fathers), shift-work schedules, cultural backgrounds, and child medical profiles. Standardized measurement batteries should combine objective actigraphy, passive digital tracking, validated feeding questionnaires, and micro-analytic video coding to ensure robust, reproducible science.

## Figures and Tables

**Table 1:** Hypothesized Pathways Linking Parental Restrictive/By-Proxy Behaviors (RBP) to Child Feeding Outcomes: Evidence Ratings, Confounders, and Competing Explanations.

Proposed Pathway/Link	Hypothesized Direction	Current Evidence Rating	Key Confounders to Control	Competing Theoretical Explanations
Link 1: Parental RBP → Sleep Loss and Fatigue	Direct positive (+)	Moderate (Derived mainly from general adult samples)	Chronotype, child night-waking, primary insomnia, shift work	Sleep loss is driven by circadian eveningness, somatic arousal, or child awakenings rather than autonomy reclamation.
Link 2a: Sleep Loss → Cognitive/Emotion Depletion	Direct positive (+)	Strong (Robust neurocognitive literature)	Parental depression, anxiety, physical health conditions	Daytime fatigue stems from underlying psychological distress or somatic illness rather than sleep restriction.
Link 2b: Depletion → Daytime Technoference	Direct positive (+)	Moderate (Observed in parent device-use studies)	Baseline smartphone addiction, habitual media habits, daily stress	Technoference is an established habitual impulse or coping mechanism for boredom rather than a depletion effect.
Link 3: Technoference → Reduced Mealtime Responsiveness	Direct negative (–)	Moderate-to-Strong (Supported by observational feeding studies)	Child temperament, maternal eating pathology, mealtime setting	Reduced responsiveness is caused by general parental withdrawal or depression rather than active device absorption.
Link 4: Reduced Responsiveness → Child Feeding Difficulties	Direct/Indirect positive (+)	Moderate-to-Strong (Validated in feeding-style literature)	Child medical conditions, sensory food sensitivities, oral-motor delays	Child feeding difficulties stem primarily from intrinsic child neurodevelopmental or sensory profiles rather than parental cue-reading.
Link 5 (Direct Chain): Parental RBP → Child Feeding Outcomes	Indirect sequential	Weak/Hypothetical (No direct empirical testing to date)	All above confounders combined	The pathway is non-existent; RBP has no unique predictive validity over general sleep loss or parental depression.

Note. RBP = restrictive/by-proxy behavior (i.e., parental restriction or regulation enacted on the child’s behalf, such as screen-time or bedtime control). Evidence ratings reflect the strength and directness of empirical support currently available for each pathway, not the strength of the hypothesized effect itself. Links 1 through 4 represent sequential mechanistic steps; Link 5 represents the full, untested indirect chain from parental RBP to child feeding outcomes.

## Data Availability

No new data were created or analyzed in this study. Data sharing is not applicable to this article.
